# Alternate-day fasting increases the risk of decreased immune cells and anemia in DSS-induced colitis mice

**DOI:** 10.3389/fnut.2026.1835068

**Published:** 2026-08-17

**Authors:** Weijie Shen, Huiru Lv, Zhen Wu, Sicheng Luo, Zixuan Liao, Zekai Xu, Li Xu

**Affiliations:** 1 Key Laboratory of Innate Immunity and Chronic Inflammatory Diseases, Jiangxi Provincial Department of Education, Key Laboratory of Prevention and Treatment of Cardiovascular and Cerebrovascular Diseases, Ministry of Education, School of Basic Medicine, Gannan Medical University, Ganzhou, China; 2 Ganzhou People’s Hospital (Southern Medical University Ganzhou Hospital / The Affiliated Ganzhou Hospital of Nanchang University), Ganzhou, Jiangxi, China; 3 First Affiliated Hospital, Gannan Medical University, Ganzhou, Jiangxi, China

**Keywords:** alternate-day fasting, anemia, IBD, immunity, intermittent fasting

## Abstract

Intermittent fasting (IF) is a widely adopted dietary strategy with well-documented benefits in the management of metabolic diseases. However, its effects in pathological settings characterized by severe tissue injury, such as inflammatory bowel disease (IBD), remain controversial. In this study, we investigated the impact of alternate-day fasting (ADF) on disease activity and immune responses in a mouse model of dextran sulfate sodium (DSS)-induced colitis. Mice were subjected to a 35-day ADF protocol, and acute colitis was induced using 3% DSS initiated 7 days prior to the conclusion of the fasting regimen. Multiple indicators were assessed, including body weight, survival rate, colonic tissue damage and barrier integrity, immune cell profile alterations in the bone marrow and spleen, as well as changes in peripheral blood cell counts. Our results showed that ADF significantly exacerbated colonic inflammation, leading to more severe body weight loss and reduced survival. Histopathological examination revealed that ADF impaired the structural integrity of the colonic mucus layer. Flow cytometric analysis further demonstrated that ADF markedly reduced specific innate immune cell populations in the bone marrow and reduced lymphocyte counts in the spleen. Furthermore, ADF-treated mice exhibited signs of anemia. Taken together, these findings indicate that, in acute DSS-induced colitis, strict ADF intervention does not alleviate intestinal injury but instead exacerbates disease progression by suppressing tissue repair, weakening systemic immune function, and promoting anemia. Therefore, the use of intermittent fasting should be carefully evaluated based on the specific pathological context, and caution is strongly recommended for patients with active inflammatory bowel disease.

## 1 Introduction

Intermittent fasting (IF), a dietary strategy alternating periods of feeding and fasting, has garnered significant attention. Currently, mainstream IF regimens include ADF, time-restricted feeding (TRF), the 5:2 diet, and the fasting-mimicking diet (FMD) (1). Extensive studies confirm its profound efficacy in ameliorating various metabolic disorders (2, 3). Substantial evidence from both animal models and human trials indicates that IF confers multidimensional health benefits against metabolic diseases, such as obesity, type 2 diabetes, and cardiovascular diseases (4–6). The biological underpinnings of these beneficial effects are complex, encompassing autophagy activation (7), mitochondrial optimization (8, 9), gut microbiota remodeling (10), and systemic inflammation suppression (11).

Inflammatory bowel disease (IBD), characterized mainly by Crohn’s disease (CD) and ulcerative colitis (UC), represents a chronic gastrointestinal inflammatory condition driven by genetic susceptibility, environmental triggers, and immune dysregulation (12). Over the past decades, the global incidence of IBD has continued to rise (13), with dietary habits widely recognized as a critical variable influencing its onset and progression (14). Specifically, the modern industrial lifestyle—characterized by high-calorie, ultra-processed diets—is closely linked to the surge in IBD prevalence (15). Given the anti-inflammatory properties of IF, it may represent a potential adjunctive therapy for IBD.

However, current research on the association between IF and IBD remains contradictory. On the one hand, multiple studies suggest that IF alleviates colitis symptoms (2, 16). For instance, experimental evidence indicates that FMD modulates gut microbiota homeostasis, promotes intestinal epithelial regeneration, and ameliorates DSS-induced intestinal injury (17). On the other hand, emerging evidence warns of potential risks in specific IBD subpopulations. A clinical study by Negm et al. involving 80 IBD patients (60 UC, 20 CD) observed disease trajectories before and after Ramadan intermittent fasting, revealing an increased risk of relapse in elderly male UC patients (18). Taken together, the effects of fasting on IBD have not been fully elucidated. Further investigations are still warranted to clarify the effects of ADF in IBD.

Most existing animal studies examining the effects of ADF on IBD utilize two prevalent protocols: either initiating the fasting intervention after the establishment of DSS-induced colitis or concurrently with DSS administration (2, 14, 16, 19). To date, there is a paucity of research evaluating the potential risks associated with long-term ADF pretreatment prior to the induction of acute colitis. In the present study, we employed ADF—one of the most widely utilized fasting regimens—on mice. Following prolonged ADF treatment, we induced an acute colitis model using standard DSS administration. Subsequently, we conducted a systematic evaluation of how this pretreatment influenced disease severity, intestinal tissue integrity, and systemic immune function. This study aim to delineate the applicable scope of dietary interventions for IBD and provide practical insights for the clinical nutritional management of IBD patients.

## 2 Materials and methods

### 2.1 Mice

All experimental animals were 8-week-old male C57BL/6 mice (Jiangsu Huachuang Xinnuo Pharmaceutical Technology Co., Ltd). The mice were allowed a one-week acclimatization period in a specific pathogen-free (SPF) environment at the Animal Experiment Center of Gannan Medical University. Throughout the entire experimental process, all mice were housed under uniform standardized environmental conditions, including controlled temperature, humidity, and a 12 h light/dark cycle. To reduce the potential confounding influence of caloric intake on our experimental outcomes, we strictly maintained consistent dietary conditions and feeding management practices between the three experimental groups. All animal experimental procedures were carried out in strict compliance with the protocols approved by the Animal Care and Use Committee of Gannan Medical University.

### 2.2 Fasting protocols in mice

The experimental fasting intervention spanned 35 days. Forty mice were randomly assigned to different groups.

- Control group (*n* = 12): mice had *ad libitum* access to food and water throughout the experiment and received no DSS.
- Control + DSS group (*n* = 14): mice were fed *ad libitum* throughout the study. Mice received 3% DSS in drinking water from day 29 to day 33, followed by regular water until day 35.
- ADF+DSS group (*n* = 14): mice were maintained on an ADF regimen throughout the experiment. They received 3% DSS in drinking water from day 29 to day 33, followed by regular water until day 35.

### 2.3 DSS-induced colitis model

This study established the acute colitis model following well-established protocols described in published literature (20–24). Briefly, C57BL/6 mice were given sterile distilled water supplemented with 3% DSS (MP Biologicals, 160110) for 5 consecutive days, followed by regular drinking water for an additional 2 days. Body weight loss, hematochezia, diarrhea and general activity of mice in each group were monitored throughout the experiment. The DSS solution was freshly prepared before use each day. To minimize variations in DSS intake among groups, the tightness and water outflow of the bottles were checked daily, and damaged devices were replaced promptly.

### 2.4 Assessment of disease activity index (DAI)

DAI is a semi-quantitative scoring system widely used to assess the severity of experimental colitis. The total score is the sum of three independent subscores, ranging from 0 to 12. The detailed scoring criteria are as follows:

**Table 1:** 

Score	Weight loss (%) (vs. initial weight)	Stool consistency	Fecal bleeding
0	No loss or gain	Normal, formed pellets	No bleeding (occult blood negative)
1	1–5	–	–
2	5–10	Loose stools (soft, not adherent to the anus)	Occult blood positive (requires test strip detection)
3	10–15	–	–
4	> 15	Diarrhea (liquid stools, adherent to the anus)	Gross bleeding (visible to the naked eye)

Calculation Formula: Total DAI Score = (Weight Loss Score) + (Stool Consistency Score) + (Fecal Bleeding Score).

### 2.5 Reverse transcription–quantitative polymerase chain reaction (RT–qPCR)

Total RNA was extracted from homogenized colon tissue using Trizol reagent. Following this, RNA was reverse-transcribed into cDNA. qPCR was then performed on an ABI QuantStudio 7 Flex system using a SYBR Green kit (Thermo Fisher Scientific, A25742) with gene-specific primers. The relative expression of target genes was calculated using the 2^–ΔΔCT^ method, with *Actb* serving as the internal reference control. The sequences of all primers used are listed in Supplementary Table 1.

### 2.6 Measurement of serum inflammatory cytokines by ELISA

Serum levels of IL-6, IL-17A, TNF-α and serum iron were determined using corresponding commercial ELISA kits (Beijing Jingmei Biotech Co., Ltd., JM-02446M1, JM-03039M1, JM-02415M1, JM-12028M1) according to the manufacturer’s instructions. Briefly, mouse blood was harvested via retro-orbital plexus puncture, allowed to clot at room temperature for 30 min, and centrifuged at 3000 × *g* for 15 min at 4 °C to obtain serum. The separated serum was aliquoted and stored at −80 °C until use. For ELISA assays, undiluted serum samples and standards (100 μL/well) were incubated in pre-coated 96-well plates for 2 h at room temperature. Following thorough washing, biotinylated detection antibody and streptavidin-HRP solution were added sequentially and incubated for 1 h and 30 min in the dark, respectively. After 15-min color development with TMB substrate, the reaction was stopped by 2 M sulfuric acid. The optical density was immediately measured at 450 nm with a reference wavelength of 620 nm using a microplate reader. Standard curves were generated via a four-parameter logistic (4-PL) model with SoftMax Pro 7.0 software. All samples were tested in duplicate, and the intra-assay coefficient of variation was controlled below 8%.

### 2.7 Hematoxylin–eosin (H&E) staining and pathological scoring

#### 2.7.1 H&E staining

Mice were euthanized via cervical dislocation. Whole colons were rapidly isolated, stripped of mesenteric tissues, and washed with ice-cold sterile PBS to eliminate luminal contents. All tissue operations were completed on ice within 10 min to maintain histological structure. The cleaned colons were flattened with mucosa facing upward and rolled from the caecum end into standard Swiss rolls with a diameter of 3–4 mm. Colon tissues were fixed in 4% neutral buffered formalin at 4 °C for 24 h. After PBS rinsing, tissues were processed for gradient dehydration, transparency and paraffin embedding by an automatic tissue processor. Paraffin-embedded tissues were cut into continuous 4 μm-thick sections. The sections were spread in a 42 °C water bath, mounted on poly-L-lysine-coated slides, and dried overnight at 37 °C. Finally, sections underwent routine deparaffinization, rehydration and H&E staining, followed by dehydration, xylene clearing and neutral balsam sealing for subsequent histological analysis.

#### 2.7.2 pathological scoring

Histopathological damage was independently evaluated and scored by two researchers who were blinded to the experimental groups using a light microscope. The scoring system integrated four parameters: inflammatory cell infiltration depth, crypt architectural damage, ulceration/mucosal erosion extent, and mucosal hyperplasia/reparative response, with the criteria as follows:

Inflammatory cell infiltration depth (0–3 points): 0 = no abnormality; 1 = mucosal-limited inflammation; 2 = inflammation extending to the submucosa; 3 = transmural inflammation or severe inflammatory aggregates.

Crypt architectural damage (0–4 points): 0 = intact crypts; 1 = loss of the basal 1/3 of crypts; 2 = loss of the basal 2/3 of crypts; 3 = total crypt loss with intact surface epithelium; 4 = complete loss of both crypts and surface epithelium (ulceration).

Ulceration/mucosal erosion extent (0–3 points): Evaluated by the area of complete epithelial loss in inflamed regions. 0 = no ulcer; 1 = single small focal ulcer (< 10 crypt widths); 2 = multiple or moderate-sized ulcers (10–50 crypt widths); 3 = extensive large or confluent ulcers (> 50 crypt widths).

Mucosal hyperplasia/reparative response (0–2 points): 0 = normal mucosal thickness without hyperplasia; 1 = mild to moderate hyperplasia; 2 = severe hyperplasia or dysplasia.

The total histological score of each section was the sum of the four sub-scores, and the final score was the average of the two independent evaluations.

### 2.8 AB-PAS staining

AB-PAS staining was used to detect acidic and neutral mucins in intestinal tissues. Paraffin sections were dewaxed and rehydrated sequentially in clearing solution, absolute ethanol, and 75% ethanol, followed by rinsing with running water. The sections were successively stained with solution C for 15 min, acidified with solution B for 15 min, and incubated with prewarmed solution A in the dark for 1 h. After a final 5-min rinse, the sections were dehydrated, cleared with xylene, and sealed with neutral balsam. Histological images were acquired under a light microscope, and ImageJ software was applied to quantify the AB-PAS-positive area and count goblet cell numbers in each field of view.

### 2.9 Immunohistochemical (IHC) analysis

For immunohistochemical staining, deparaffinized and rehydrated tissue sections underwent antigen retrieval via microwave irradiation, followed by incubation with the following primary antibodies: anti-MUC2 (Abcam, ab272692), anti-OCCLUDIN (CST, 91131s) and anti-ZO-1 (Proteintech, 21773-1-AP). The staining results were quantitatively analyzed using ImageJ software, with the integrated density and percentage of positive area used to represent target protein expression levels. For each group, 6 mice were included, and 4 non-overlapping fields per mouse were examined. Data were visualized and graphed using GraphPad Prism 11 software, and statistical significance was analyzed with the same software.

### 2.10 Western blot

Western blot analysis was performed to detect MUC2 protein expression in mouse colonic tissues. Briefly, total protein was extracted using RIPA lysis buffer, quantified via BCA assay, and separated by 10% SDS-PAGE. Proteins were transferred onto PVDF membranes, blocked with 5% BSA, and incubated overnight at 4 °C with anti-MUC2 primary antibody (Abcam, ab272692), followed by HRP-conjugated secondary antibody. Blots were visualized using enhanced chemiluminescence substrate and imaged with a ChemiDoc system. β-actin (Proteintech, 66009-1-lg) was used as an internal loading control.

### 2.11 Flow cytometry

Single-cell suspensions were prepared from the bone marrow and spleen of mice from each group through mechanical dissociation. All samples were analyzed using a BD FACS Canto II flow cytometer. Cell staining was performed according to the manufacturer’s protocols with the following fluorescently conjugated antibodies: anti-CD45 (30-F11), anti-CD3 (17A2), anti-CD4 (GK1.5), anti-CD8a (53–6.7), anti-CD11b (m1/70), anti-CD19 (6D5), anti-Ter119 (Ter-119), anti-MHCII (M5/114.15.2), and anti-F4/80 (BM8), all antibodies purchased from Biolegend.

### 2.12 Routine blood analysis

Peripheral blood (150  μL) was collected from mice in each experimental group. Prior to collection, an anticoagulant solution was prepared by mixing 100 μL of EDTA (Beyotime Biotechnology, ST066) with 50 μL of PBS. A standard five-part differential hematological analysis was subsequently performed to determine red blood cell counts, hemoglobin concentration and hematocrit levels.

### 2.13 Statistical analysis

All statistical analyses were conducted via GraphPad Prism 11. Data were expressed as mean ± SEM. One-way ANOVA with Newman–Keuls *post-hoc* test was used for intergroup multiple comparisons. Kaplan–Meier survival curves were plotted, and log-rank (Mantel–Cox) test was applied for survival comparison. Median survival time and 95% confidence intervals (CIs) were calculated when appropriate. Multivariate Cox proportional hazards regression was performed to evaluate the effects of multiple covariates on survival outcomes, with hazard ratios and 95% CIs calculated accordingly. All tests were two-tailed, and *p* < 0.05 was defined as statistically significant.

## 3 Results

### 3.1 ADF reduces body weight and survival rate in DSS-induced colitis mice

**Figure 1:**
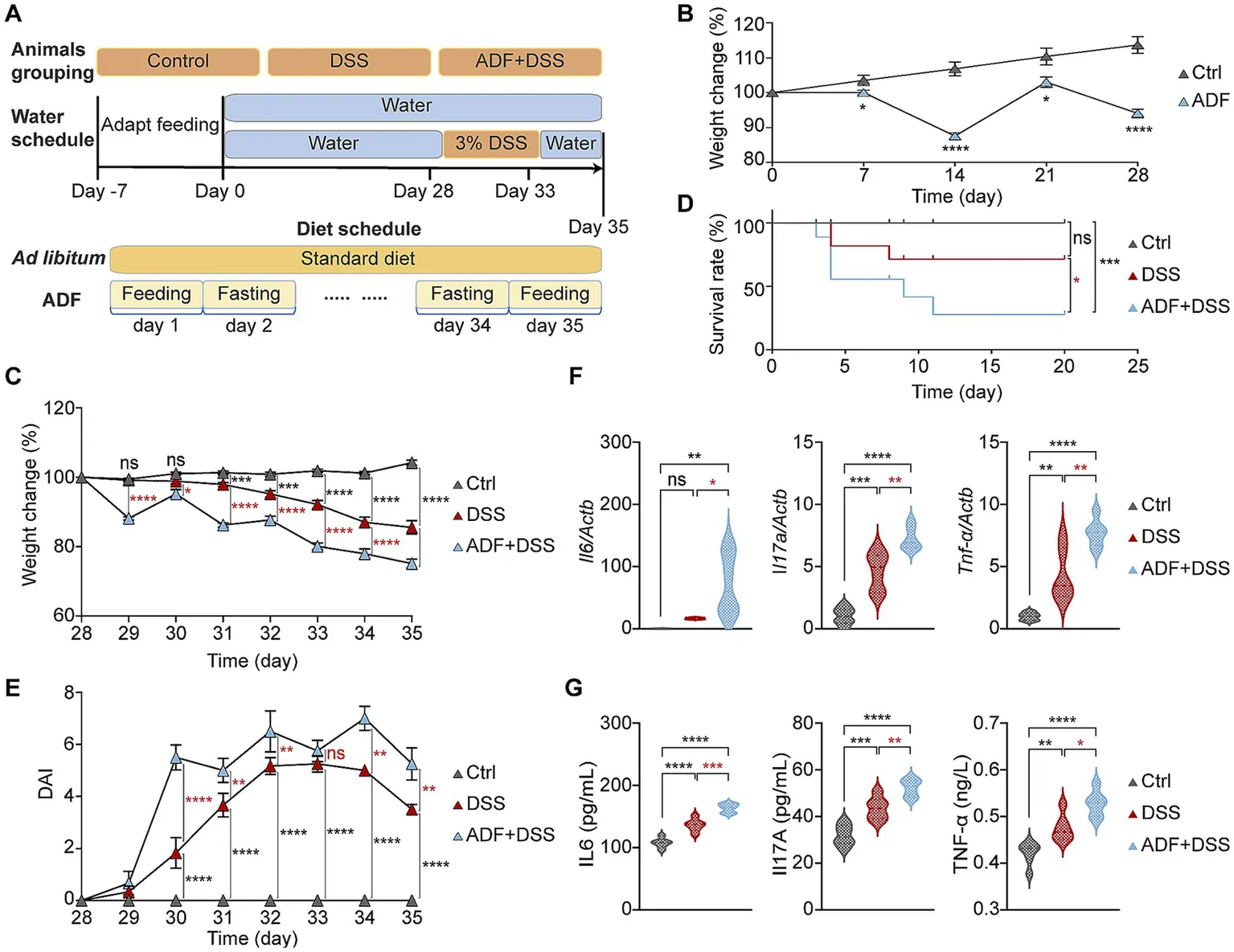
Effects of ADF on the weight, survival rates, DAI scores and inflammatory factors in DSS-treated mice. **(A)** Experimental schedule of animal treatments; timeline depicting the diet of *Ad* libitum and ADF; **(B)** Weight change of mice in each group mice in the first 28 days. The black stars represent the difference between ADF and Ctrl group mice (Ctrl *n* = 12; ADF *n* = 14); **(C)** Weight change of mice in each group from day 29 to day 35, black asterisks: Control vs. DSS group; red asterisks: DSS vs. ADF+DSS group (Ctrl *n* = 12; DSS, ADF+DSS *n* = 14); **(D)** Effects of ADF on the survival rates in mice. Survival rate of mice in each group mice from day 28 to day 48 (Ctrl *n* = 6; DSS, ADF+DSS *n* = 8); **(E)** The DAI scores of mice in each group. The black stars represent the difference between Ctrl and DSS group. The red stars represent the difference between ADF+DSS and DSS group (Ctrl *n* = 12; DSS, ADF+DSS *n* = 14); **(F)** Using RT-qPCR to detect the expression of inflammatory factors in the colonic tissues of mice from each group mice (*n* = 6/group); **(G)** Measurement of serum inflammatory cytokine levels by ELISA (*n* = 6/group). Data are representative of at least three independent experiments. Survival curves were generated using the Kaplan–Meier method. Comparisons of survival distributions between groups were performed using the log-rank (Mantel–Cox) test. Median survival times and 95% confidence intervals (CIs) were calculated for each group where applicable. Data are presented as mean ± SEM and statistical significance was determined by one-way ANOVA with Newman–Keuls multiple comparisons test, *p < 0.05, **p < 0.01, ***p < 0.001, ****p < 0.0001, ns: No significant difference.

Figure 1A provides a schematic representation of the experimental design. Mice were randomly assigned to one of three groups: the Control group, the DSS group and the ADF+DSS group. Animal modeling was conducted over a 35-day period. The detailed experimental regimens are presented in Materials and Methods 2.2 and 2.3. Following the completion of the experimental modeling, we evaluated a range of physiological parameters across all groups. As expected, compared with the Control group, mice in the DSS group showed a marked shortening of colon length (Supplementary Figure 1), which was consistent with typical pathological manifestations of DSS-induced acute colitis reported in previous studies. In addition, we further monitored dynamic changes in mouse body weight during the whole experiment. The results revealed that mice in the single ADF group had significantly decreased body weight at day 7 (*p* < 0.05), day 14 (*p* < 0.0001), day 21 (*p* < 0.05), and day 28 (*p* < 0.0001) relative to the Control group (Figure 1B), suggesting that ADF may effectively reduce body weight under basal conditions.

Subsequently, we investigated the impact of ADF on mice with DSS-induced colitis. First, mice in the ADF+DSS group consistently exhibited lower body weight over a period of seven consecutive days compared to those in the DSS group (Figure 1C). In addition, survival analysis demonstrated that ADF markedly reduced the survival rate of mice with DSS-induced colitis: the survival rate was 62.5% in the DSS group, whereas it dropped to only 25% in the ADF+DSS group (Figure 1D). Furthermore, the disease activity index (DAI) was significantly higher in the ADF+DSS group than that in the DSS group (Figure 1E). Collectively, in this mouse model, ADF significantly reduced body weight and survival rate, while substantially elevating the DAI.

### 3.2 ADF may potentially compromise colonic tissue integrity and exacerbate inflammation in mice with DSS-induced colitis

**Figure 2:**
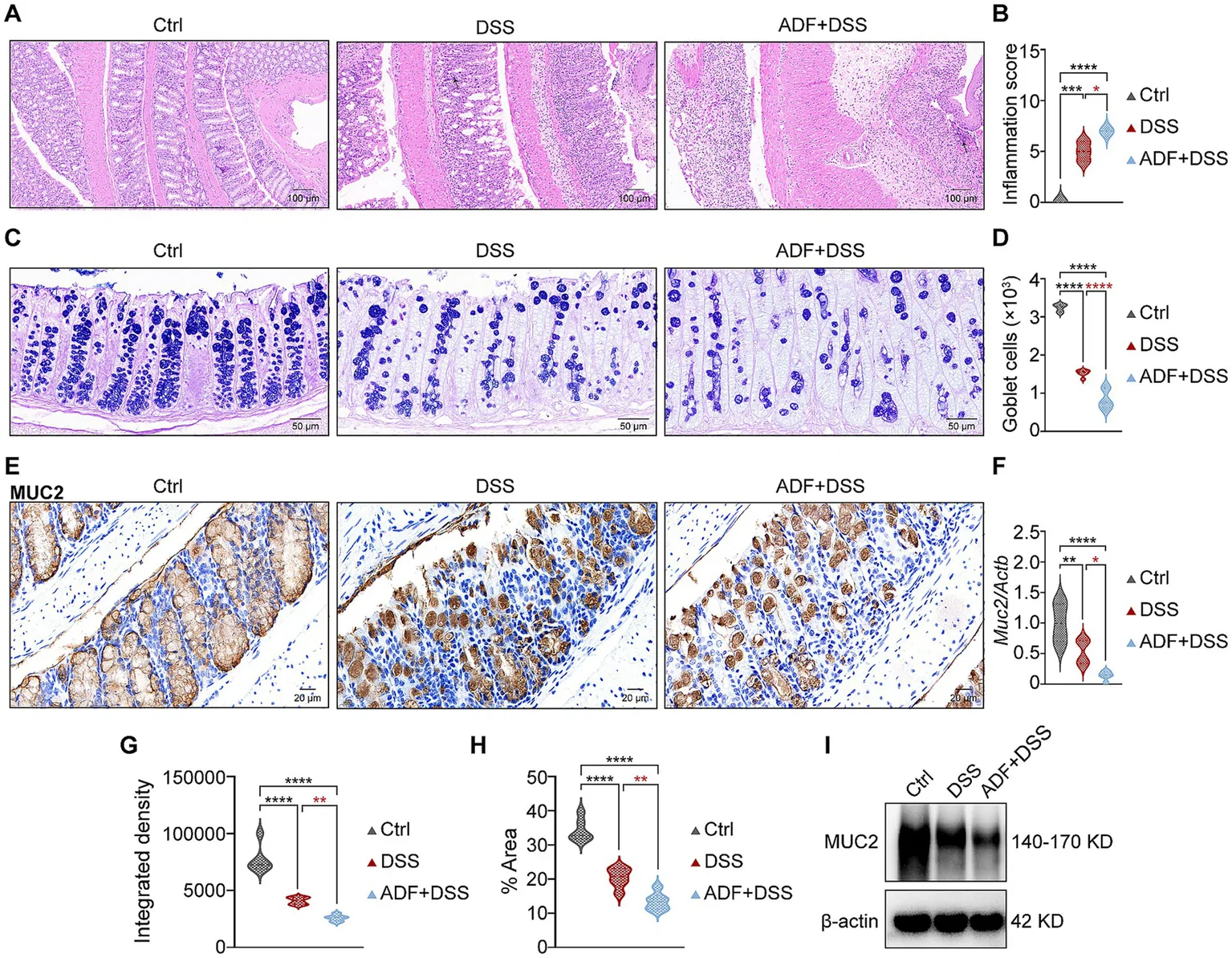
Effects of ADF on colon tissue pathology and MUC2 expression in DSS-treated mice. **(A)** Representative images of H&E-stained colonic tissues. Black arrows indicate areas of inflammatory cell infiltration. Scale bar = 100 μm (*n* = 3/group). **(B)** Inflammation score results of H&E staining in colon tissue of mice in each group (*n* = 3/group). **(C,D)** Representative images of AB-PAS-stained colonic sections and the number of goblet cells. Scale bar = 50 μm (*n* = 6). **(E)** Immunohistochemistry was used to detect the expression of MUC2 in colon tissue of each group mice. Scale bar = 20 μm (*n* = 6/group). **(F)** RT-qPCR was used to detect the expression of *Muc2* in colon tissue of mice in each group (*n* = 6/group). **(G)** Statistical analysis of IHC results. The bar graph shows the integrated density of the MUC2 protein expression (*n* = 6/group). **(H)** Quantitative analysis of IHC staining. The %Area was measured to assess the MUC2 protein expression level (*n* = 6/group). **(I)** Western blot analysis was performed to detect the expression of MUC2 protein in mice from each experimental group. Data are representative of at least three independent experiments. Data are presented as mean ± SEM and statistical significance was determined by one-way ANOVA with Newman–Keuls multiple comparisons test, *p < 0.05, **p < 0.01, ***p < 0.001, ****p < 0.0001, ns: No significant difference.

Intestinal crypts, which are specialized glandular structures composed of goblet cells, Paneth cells, and intestinal stem cells, play a crucial role in maintaining normal intestinal physiological function (25). To assess the impact of ADF on colonic tissue integrity and the extent of inflammatory infiltration in DSS-induced colitis, we conducted H&E staining and histological scoring on mouse colon sections. Histological analysis indicated that, compared to the control group, DSS-treated mice exhibited colonic inflammatory infiltration extending into the mucosal layer, aligning with the characteristic pathological features of the DSS colitis model. Importantly, in contrast to the DSS group, mice subjected to the ADF+DSS regimen demonstrated inflammatory infiltration reaching the submucosal layer (Figure 2A), along with a significantly elevated histological inflammation score (Figure 2B; Supplementary Table 2).

To further elucidate the pro-inflammatory effects of ADF, we assessed cytokine expression in colonic tissues utilizing RT-qPCR. The results showed that compared with the DSS group, the ADF+DSS treatment significantly upregulated the mRNA levels of pro-inflammatory cytokines, including interleukin-6 (*Il6*), interleukin-17A (*Il17a*), and tumor necrosis factor-*α* (*Tnf-α*) (Figure 1F), suggesting that ADF intensifies local intestinal inflammation. Correspondingly, ELISA analysis of serum cytokines revealed that the circulating levels of IL-6, IL-17A, and TNF-α were also markedly elevated in the ADF+DSS group compared to the DSS group (Figure 1G). These findings corroborate the observations in colonic tissues and indicate that ADF may exacerbate both local and systemic inflammatory responses in DSS-induced colitis.

### 3.3 ADF may lead to a reduction in goblet cell numbers and *Muc-2* expression, while having minimal impact on the expression of tight junction proteins *Occludin*, *TJP1* and *Claudin 1* in colitis induced by DSS

DSS-induced colitis is frequently linked to a marked impairment of intestinal barrier function. To systematically evaluate the influence of ADF on the integrity of the intestinal barrier, we analyzed the expression levels of mucin-2 (*Muc-2*) and tight junction proteins, including *Claudin 1*, *TJP1* and *Occludin* in the colonic tissues of mice across different groups. Compared to the DSS group, the ADF+DSS group exhibited aggravated damage to the colonic mucus barrier, as indicated by a significant reduction in goblet cell numbers (Figures 2C,D) and a notable downregulation of MUC-2 protein expression (Figures 2E–I). Notably, under the current experimental conditions, ADF may not significantly alter the expression levels of the tight junction proteins *Claudin 1* (Supplementary Figure 2A), *TJP1* (Supplementary Figures 2B,G) and *Occludin* (Supplementary Figures 2C–F). Our subsequent research will focus on examining the regulatory effects of ADF on additional tight junction proteins, with the aim of further elucidating its roles and underlying molecular mechanisms in maintaining intestinal barrier integrity.

### 3.4 ADF may decrease the abundance of certain immune cell populations in mice with DSS-induced colitis

**Figure 3:**
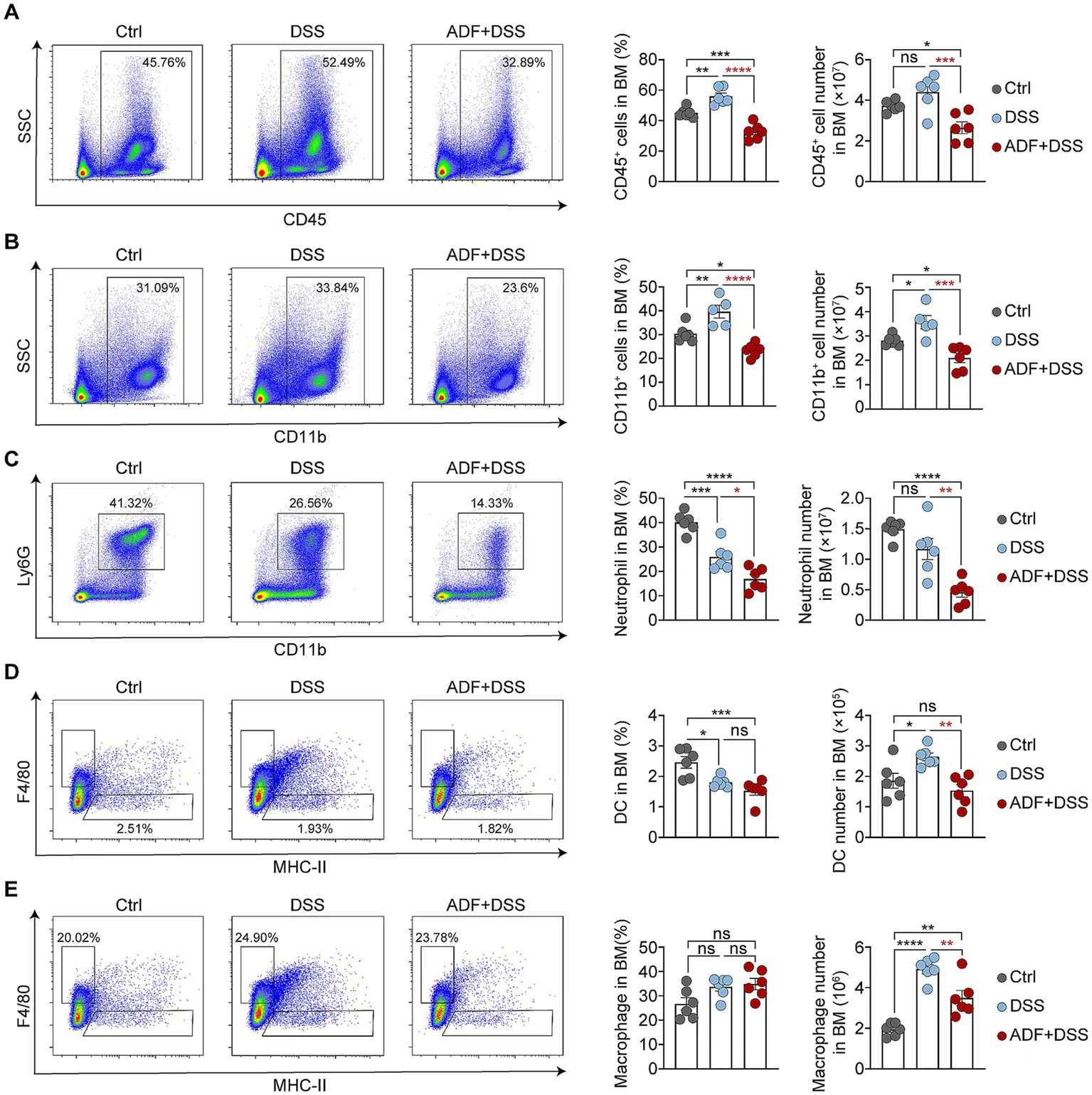
Effects of ADF on white blood cells (WBC), myeloid cells, neutrophils (Neu), dendritic cells (DC), and macrophages in mouse bone marrow (BM) of DSS-treated mice. **(A)** Flow cytometric quantification and statistical analysis of the number of BM WBC (CD45^+^). On the left, the flow representative diagram; In the middle, the flow result counts the cell proportion; On the right, the flow results count the number of cells (Ctrl, DSS, ADF+DSS *n* = 5); **(B)** The number of myeloid cells (CD11b^+^) in BM of mice in each group was detected by flow cytometry. The myeloid cells were labeled with CD11b antibody. Left panel, representative flow cytometry density plots. Middle and right panel, quantitative and statistical analysis (DSS *n* = 5; Ctrl, ADF+DSS *n* = 6); **(C)** Flow cytometric quantification and statistical analysis of the number of BM neutrophils (CD45^+^CD11b^+^LY6G^+^). On the left, the flow representative diagram; In the middle, the flow result counts the cell proportion; On the right, the flow results count the number of cells (*n* = 6/group); **(D)** The number of DC (CD45^+^CD11b^+^LY6G^−^MHC-II^+^F4/80^−^) in BM of mice in each group was detected by flow cytometry. Left panel, representative flow cytometry density plots. Middle and right panel, quantitative and statistical analysis (*n* = 6/group); **(E)** Quantitative and statistical analysis of BM macrophages (CD45^+^CD11b^+^LY6G^−^MHC-II^−^F4/80^+^). Left panel, representative flow cytometry density plots. Middle and right panels, quantitative and statistical analysis (*n* = 6/group). Data are representative of at least three independent experiments. Data are presented as mean ± SEM and statistical significance was determined by one-way ANOVA with Newman–Keuls multiple comparisons test, *p < 0.05, **p < 0.01, ***p < 0.001, ****p < 0.0001, ns, no significant difference.

Immune cells are crucial in the initiation and progression of IBD. The bone marrow serves as the major site where these cells are generated, differentiated and matured. To assess the potential impact of ADF on immune function in this context, we quantified various immune cell populations within the bone marrow. Leukocytes are key immune cells located in the bone marrow, and alterations in their proportion and composition can indicate changes in the organism’s systemic immune status. Our findings demonstrated a significant reduction in both the percentage and absolute count of leukocytes in the ADF+DSS group compared to the DSS group (Figure 3A). This preliminary finding suggests that ADF may disrupt hematopoietic homeostasis in colitic mice.

To further delineate the specific leukocyte subsets modulated by ADF, we conducted an investigation into its effects on myeloid cells. As previously documented, CD11b serves as a canonical marker for myeloid cells. Our findings revealed a significant reduction in both the percentage and absolute number of CD11b^+^ myeloid cells in the ADF+DSS group compared to the DSS group (Figure 3B). To further elucidate the precise myeloid subsets influenced by ADF, we quantified neutrophils, monocytes/macrophages and dendritic cells using flow cytometry. The gating strategy employed for this analysis is detailed in Supplementary Figure 3. Consistently, both the proportion and absolute number of neutrophils were markedly diminished in the ADF+DSS group relative to the DSS group, with dendritic cells and macrophages also exhibiting a decreasing trend (Figures 3C–E). Collectively, these findings suggest that among the myeloid cells in the bone marrow of colitic mice, neutrophils are the subset most significantly impacted by ADF, followed by dendritic cells and macrophages.

In contrast to the myeloid populations, we also sought to explore the effects of ADF on bone marrow lymphoid cells. For the purpose of experimental detection, these cells were identified using anti-CD3 and anti-CD19 antibodies respectively. The results demonstrated no significant differences in the numbers of bone marrow T cells and B cells between the ADF+DSS group and the DSS group, as shown in Supplementary Figures 4A,B. Collectively, these findings may suggest that ADF does not exert a noticeable impact on bone marrow T and B lymphocytes during the progression of DSS-induced colitis.

**Figure 4:**
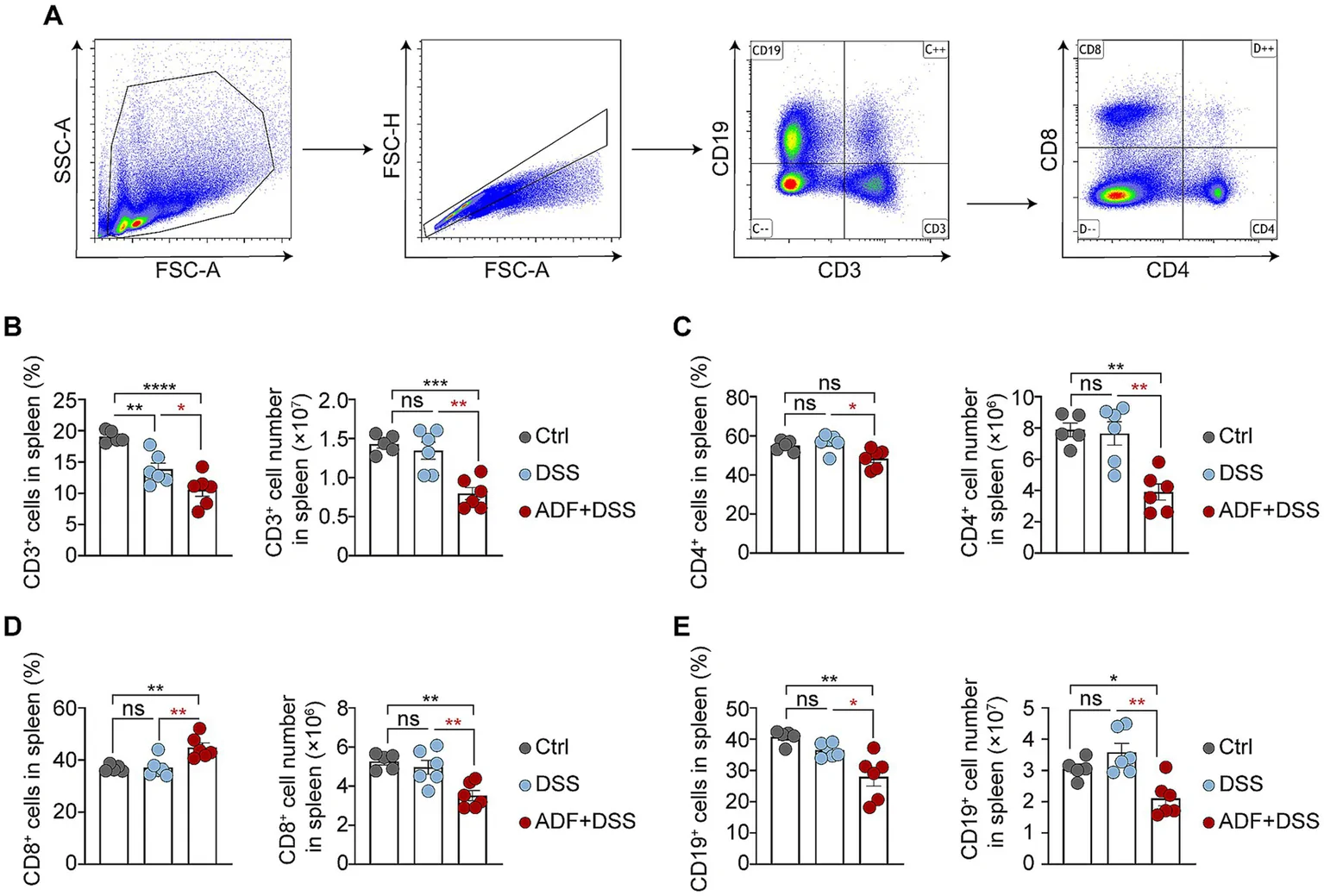
Effects of ADF on lymphocytes in the spleen of DSS-treated mice. **(A)** Representative flow cytometric analysis plot of T cell and B cells in the spleen of DSS-treated mice. CD3 antibody labeled T cells. CD19 antibody labeled B cells. CD3 and CD4 antibody labeled helper T cells (CD3^+^CD4^+^). CD3 and CD8 antibody labeled cytotoxic T lymphocytes (CD3^+^CD8^+^). **(B)** The number of T cells in the spleen of each group mice was detected by flow cytometry. On the left, the CD3^+^ cell ratio was counted. On the right, statistical results of CD3^+^ cell number (Ctrl *n* = 5; DSS, ADF+DSS *n* = 6). **(C)** Flow cytometry was used to detect the number of helper T cells in the spleen of each group mice. Left, the CD4^+^/CD3^+^ cell ratio was counted. Right, statistical results of CD3^+^CD4^+^ cell number (Ctrl *n* = 5; DSS, ADF+DSS *n* = 6). **(D)** Flow cytometry was used to detect the number of cytotoxic T lymphocytes in the spleen of each group mice. Left, the CD8^+^/CD3^+^ cell ratio was calculated. Right, statistical results of CD3^+^CD8^+^ cell number (Ctrl *n* = 5; DSS, ADF+DSS *n* = 6). **(E)** FCM detection of the number of B lymphocytes in the spleen of each group mice. On the left, the cell ratio CD19^+^/living cells was counted. On the right, statistical results of CD19^+^ cell number (Ctrl *n* = 5; DSS, ADF+DSS *n* = 6). Data are representative of at least three independent experiments. Data are presented as mean ± SEM and statistical significance was determined by one-way ANOVA with Newman–Keuls multiple comparisons test, *p < 0.05, **p < 0.01, ****p < 0.0001, ns: no significant difference.

However, the spleen is a critical site for the residence and activation of T and B lymphocytes. Therefore, we quantified T and B lymphocytes in the spleen. As depicted in Figure 4A, the representative gating strategy for flow cytometric analysis is presented. Notably, in comparison to the DSS group, the ADF+DSS group exhibited significant reductions in the percentage and absolute count of splenic CD3^+^ T cells, CD3^+^CD4^+^ helper T cells, CD3^+^CD8^+^ cytotoxic T cells and CD19^+^ B cells (Figures 4B–E). Consequently, these results may indicate that ADF has pronounced detrimental effects on T and B lymphocytes in the spleen of colitic mice.

In conclusion, ADF significantly reduces the abundance of various immune cells in the bone marrow and spleen of mice with DSS-induced colitis. Notably, under conditions of DSS-induced inflammation, ADF may increase the risk of immune dysregulation and immune exhaustion *in vivo*.

### 3.5 ADF appears to heighten the risk of anemia in mice with DSS-induced colitis

**Figure 5:**
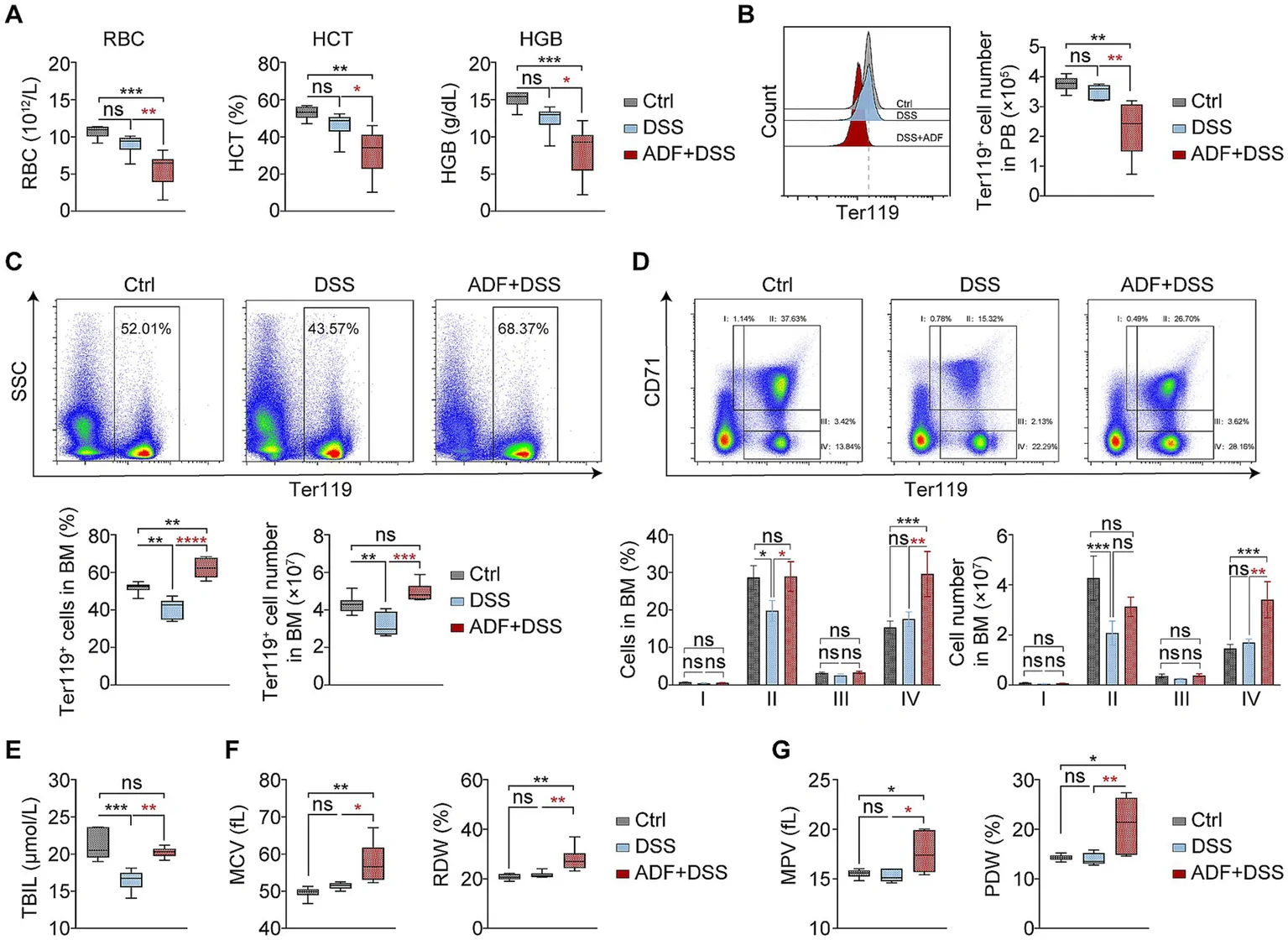
Effects of ADF on red blood cells (RBCs) and platelets in peripheral blood of DSS-treated mice. **(A)** Peripheral blood RBC, HCT and HGB in each group mice were analyzed using an automated hematology analyzer (*n* = 6/group). **(B)** Flow cytometric quantification and statistical analysis of the number of peripheral Ter119^+^ RBC of each group mice. Left panel, representative flow cytometry density plots. Right panel, quantitative and statistical analysis (*n* = 6/group). **(C)** The number of Ter119^+^ RBC in the BM of each group mice was detected by flow cytometry. Up panel, representative flow cytometry density plots. Down panel, quantitative and statistical analysis (*n* = 6/group). **(D)** Flow cytometric analysis of erythroid cells at different developmental stages in the bone marrow of each group mice. Up: Representative flow plots. Down: Relative proportions of each stage and absolute counts of each stage. BM: Bone marrow; I: Proerythroblasts (Ter119^med^CD71^hi^); II: Basophilic erythroblasts (Ter119^hi^CD71^hi^); III: Polychromatic erythroblasts (Ter119^hi^CD71^med^); IV: Orthochromatic erythroblasts (Ter119^hi^CD71^−^) (*n* = 6/group). **(E)** The level of total bilirubin (TBIL) in the serum of each group mice was measured using ELISA (*n* = 6/group). **(F)** Mean corpuscular volume (MCV) and red cell distribution width (RDW) were detected by blood cell analyzer (*n* = 6/group). **(G)** Mean platelet volume (MPV) and platelet distribution width (PDW) were detected by routine blood test (*n* = 6/group). Data are representative of at least three independent experiments. *p < 0.05, **p < 0.01, ***p < 0.001, ****p < 0.0001, ns, no significant difference.

Anemia is a prevalent and clinically significant complication in this model. To investigate whether ADF exacerbates anemia, we assessed peripheral red blood cell (RBC) count, hemoglobin (HGB) concentration, and hematocrit (HCT). Routine blood analyses demonstrated significant reductions in RBC count, HGB levels, and HCT in the ADF+DSS group compared to the DSS group (Figure 5A), suggesting that ADF may increase the risk of anemia in this context. Consistent with these findings, flow cytometry results corroborated a substantial decrease in erythrocyte numbers in the ADF+DSS group relative to the DSS group (Figure 5B). Overall, ADF may enhance susceptibility to anemia under DSS-induced colitis conditions.

To elucidate the potential mechanism underlying ADF-induced erythrocyte reduction, we investigated hemoglobin degradation metabolism. The breakdown of hemoglobin from senescent or damaged erythrocytes results in the production of substantial amounts of bilirubin. Consequently, serum bilirubin levels serve as a partial indicator of the rate of hemoglobin catabolism, as well as the liver’s capacity for bilirubin metabolism and excretion. Based on this premise, we measured serum total bilirubin levels. Our findings revealed that serum total bilirubin levels were significantly elevated in the ADF+DSS group compared to the DSS group (Figure 5E), indicating that ADF may enhance the turnover and degradation of senescent and damaged erythrocytes under inflammatory conditions.

Given that peripheral erythrocyte loss typically induces endogenous hematopoietic compensation, which usually manifests as compensatory erythropoiesis in the bone marrow, we sought to determine whether this compensatory mechanism was activated in our model. We assessed erythrocyte abundance within the bone marrow and identified a significant elevation in erythrocyte counts in the bone marrow of the ADF+DSS group relative to the DSS group (Figure 5C). This observation suggests that ADF stimulates compensatory hematopoiesis in the bone marrow of mice treated with DSS. To further elucidate the dynamic alterations in erythroid differentiation during this compensatory process, we conducted flow cytometric analysis utilizing CD71 and Ter119 markers to characterize erythroid development. Erythroid cells were categorized into four developmental stages based on CD71 and Ter119 expression: proerythroblasts (Stage I), basophilic erythroblasts (Stage II), polychromatic erythroblasts (Stage III) and orthochromatic erythroblasts (Stage IV). In comparison to the DSS group, the ADF+DSS group exhibited significantly elevated proportions and absolute numbers of basophilic erythroblasts (Stage II) and orthochromatic erythroblasts (Stage IV) within the bone marrow (Figure 5D). These findings indicate that ADF may enhance the compensatory proliferation of basophilic and orthochromatic erythroblasts in the bone marrow of mice with DSS-induced colitis.

In accordance with the aforementioned alterations in bone marrow hematopoiesis, newly formed erythrocytes resulting from compensatory erythropoiesis typically exhibit an increased cell volume, leading to an elevated mean corpuscular volume (MCV). The simultaneous presence of normal mature erythrocytes and larger reticulocytes contributes to increased heterogeneity in erythrocyte volume, which is reflected by a significant rise in red cell distribution width (RDW). Consequently, to substantiate the activation of compensatory hematopoiesis from the perspective of peripheral blood erythrocyte morphology, we measured MCV and RDW. Both parameters were significantly elevated in the ADF+DSS group compared to the DSS group (Figure 5F), thereby corroborating that ADF stimulates bone marrow compensatory erythropoiesis and promotes the release of immature erythrocytes into the peripheral circulation.

Considering that erythrocytes and platelets originate from common megakaryocyte-erythroid progenitor cells during hematopoietic development, we extended our investigation to examine platelet profiles. In light of the substantial reduction in erythrocytes induced by ADF, we further assessed changes in platelet (PLT) counts and morphological parameters. Peripheral platelet count, mean platelet volume (MPV), and platelet distribution width (PDW) were assessed. Notably, no significant differences in platelet count were detected between the ADF+DSS and DSS groups (Supplementary Figure 5B). However, both MPV and PDW were significantly elevated in the ADF+DSS group (Figure 5G). This concurrent increase in MPV and PDW may suggest enhanced bone marrow thrombopoiesis and the release of larger, newly formed platelets.

Collectively, these findings indicate that ADF may disrupt hematopoietic homeostasis under inflammatory conditions, thereby increasing the risk of anemia in mice with DSS-induced colitis. Mechanistically, the organism appears to initiate compensatory hematopoiesis in response to anemia, as evidenced by increased bone marrow erythrocytes and elevated MCV, RDW, MPV and PDW.

## 4 Discussion

This study provides preliminary insights into the potential effects of preventive ADF on intestinal injury, intestinal barrier integrity, central and peripheral immune homeostasis, and colitis-associated anemia in mice with DSS-induced acute colitis. The *in vivo* experimental findings collectively indicate that, under the current experimental conditions, preventive ADF appears to exacerbate colonic inflammatory injury. This is accompanied by disrupted immune homeostasis in the bone marrow and spleen, as well as worsened colitis-associated anemia. In conjunction with previous studies, this paper provides an objective comparison of the experimental design differences between the present study and earlier research on dietary interventions. It carefully analyzes the potential mechanisms underlying the phenotypic changes induced by ADF intervention and discusses the limitations of the current study.

Most existing preclinical animal studies have shown that appropriate dietary interventions can exert potential therapeutic effects on active colitis. The majority of mainstream research in this domain employs a therapeutic intervention paradigm, wherein fasting is initiated after acute colitis is established and intestinal inflammation stabilizes in DSS-challenged mice, to assess the alleviating effects of dietary intervention on established intestinal inflammation (17, 26). In contrast to the therapeutic regimens commonly employed in previous studies, the current research adopts a preventive intervention paradigm, wherein mice were subjected to continuous ADF prior to DSS modeling. The discrepancies in intervention timing and scheduling are likely the primary factors contributing to the divergent outcomes observed between this study and prior literature. These two intervention models exhibit substantial differences in their research focus and application scenarios. Therapeutic intervention primarily aims to alleviate and repair established intestinal inflammatory damage, thereby serving as a more accurate representation of adjuvant interventions for clinically diagnosed patients with IBD. Conversely, preventive intervention targets the pre-inflammatory stage, with the objective of modulating the basal intestinal microenvironment and immune status in advance through dietary manipulation, thereby delaying disease progression. This approach is particularly relevant for research focused on disease prevention and recurrence control in high-risk populations. Additionally, preventive intervention offers distinct experimental advantages. Firstly, the approach significantly reduces confounding interference resulting from *in vivo* microenvironmental disorders following the onset of inflammation, thereby providing an objective reflection of the early regulatory effects of diet on the intestinal barrier and gut microbiota. Secondly, it offers experimental evidence supporting early dietary prevention strategies against IBD, which holds substantial public health implications. Currently, therapeutic strategies for IBD predominantly emphasize the management of active disease episodes, rather than the prevention of disease onset or recurrence. This study aimed to evaluate the potential of ADF pretreatment as a nutritional intervention to influence the susceptibility of high-risk populations to colitis. This was achieved by modulating the basal intestinal microenvironment, encompassing the intestinal microbiota, intestinal barrier function, and immune status. We posit that this preventive research approach may offer a novel direction for the prevention and management of IBD.

A growing body of evidence suggests that IF enhances intestinal homeostasis and mitigates colitis-induced injury through multiple pathways, with its protective mechanisms being progressively validated in numerous studies. Caloric restriction has been shown to enhance autophagy in intestinal epithelial cells, modulate bile acid metabolism and intestinal stem cell function, and exert beneficial effects on the repair of intestinal damage (27). IF promotes the nuclear translocation of transcription factor EB, which regulates autophagy and mitochondrial fusion, thereby alleviating DSS-induced intestinal inflammatory injury (28). Furthermore, a FMD has been found to reshape gut microbiota, promote intestinal tissue repair, and mitigate the pathological lesions associated with inflammatory bowel disease (17). Furthermore, IF has been shown to downregulate the expression of inflammation-related factors, including *Il-1β* and *Igf-I,* thereby exerting anti-inflammatory effects (2, 14, 16, 29). This dietary approach also alters the composition of the gut microbiota and ameliorates intestinal microecological imbalances to some extent (3, 26). Intestinal stem cells are identified as pivotal cellular targets mediating the intestinal protective effects of caloric restriction (17, 30). However, our findings indicate that preventive ADF exacerbates colonic inflammation. In light of the reported protective mechanisms of fasting, we propose a preliminary hypothesis for further investigation: preventive ADF may result in an inadequate local nutrient supply in the colon. Consequently, intestinal stem cells and colonic epithelial cells may be deprived of essential repair substrates, such as glutamine and short-chain fatty acids (SCFAs), thereby impairing the intrinsic repair capacity of the intestinal barrier. Upon subsequent DSS-induced inflammatory challenge, intestinal damage may be exacerbated. Future studies should aim to quantify the levels of key intestinal metabolites, such as glutamine and SCFAs, to further validate this hypothesis. Furthermore, direct experimental evidence is still lacking to clarify whether dysregulated autophagy and gut microbiota dysbiosis contribute to this adverse phenotype, which remains to be elucidated in future studies.

To further elucidate the specific mechanisms by which ADF influences intestinal barrier function, we investigated markers associated with both the intestinal mucus and physical barriers. Previous research has indicated that fasting interventions can enhance the structure of intestinal tight junctions and maintain the integrity of the intestinal physical barrier under certain conditions (17, 26). Nonetheless, our current study did not observe significant alterations in the overall expression levels of tight junction proteins, such as *Occludin*, *TJP1* and *Claudin-1*, following ADF treatment. Instead, we identified a trend indicating impairment of the mucus barrier, evidenced by a reduction in goblet cell numbers and decreased *Muc2* expression (Figure 2; Supplementary Figure 2). Based on our experimental design and existing literature, we hypothesize that the discrepancies in findings may be attributed to three primary factors. Firstly, fasting regimens differ considerably, and various fasting patterns elicit distinct adaptive responses in the intestine. Zhang et al. compared multiple fasting protocols and found that time-restricted feeding and intermittent energy restriction alleviated intestinal barrier damage, whereas ADF failed to exert obvious protective effects (26), which is partially consistent with our observations. Furthermore, the timing of the intervention in our study diverges from that in previous investigations, and such variations in experimental protocols likely constitute the primary reason for the observed inconsistencies in results. Secondly, there are differences in the baseline conditions and sampling time points. Most studies reporting the protective effects of fasting on tight junctions have been conducted using animal models with pre-existing pathological damage to the intestinal barrier, where fasting primarily served to repair established injuries (31–33). In contrast, the interventions in our study were implemented when the intestinal barrier was structurally intact and without prior inflammatory damage, which may account for the lack of significant alterations in the expression of tight junction proteins due to ADF. Thirdly, the indicators assessed in this study have certain limitations. The functionality of tight junctions is influenced not only by the total protein abundance but also by protein localization, complex assembly, phosphorylation modifications, and other regulatory processes. Thus, measuring total protein expression alone may not fully capture the subtle potential effects of ADF on tight junction function. In conclusion, our preliminary findings suggest that ADF may selectively influence the intestinal barrier within this preventive intervention framework, primarily compromising the mucus barrier. This impairment may be associated with an increased vulnerability to intestinal inflammation in mice.

Beyond local changes in the intestinal barrier, this study also investigated the potential effects of ADF on systemic immune homeostasis. Immune imbalance is a pivotal factor in the onset and progression of IBD. As key immune organs, the bone marrow and spleen collaboratively regulate the development, differentiation, and immune responses of immune cells. Our results indicate that the proportions of several immune cell subsets in the bone marrow and spleen were reduced to varying extents following ADF intervention, suggesting a potential disruption of immune homeostasis by ADF. However, the specific regulatory pathways responsible for this reduction remain to be clarified. Previous studies have shown that intermittent fasting influences the proliferation and differentiation of immune cells through the PI3K/Akt/mTOR nutrient-sensing pathway (34). In the interim, fasting-induced stress can moderately increase systemic glucocorticoid levels, subsequently triggering lymphocyte apoptosis and cellular redistribution (35–37). Both mechanisms may contribute to the immunophenotypic alterations observed in this study. Nonetheless, the current experimental data are insufficient to delineate their individual contributions, necessitating further research to substantiate these hypotheses.

Anemia is a prevalent extraintestinal complication of IBD and can impede overall disease recovery in patients. Our findings indicated that mice subjected to ADF intervention exhibited more pronounced anemic phenotypes compared to those receiving DSS treatment alone, suggesting that ADF may exacerbate colitis-associated anemia. Drawing from the existing literature on iron metabolism and our experimental data, we hypothesize that this phenomenon is mediated by multiple interacting factors. During the progression of colitis, the body’s demand for iron increases, and persistent iron loss occurs due to hematochezia (38–41). On one hand, cyclic fasting under ADF reduces exogenous iron intake. Consistently, serum iron levels were diminished in the ADF+DSS group (Supplementary Figure 5A), suggesting that insufficient iron intake is one contributor to aggravated anemia. On the other hand, ADF was observed to slightly upregulate the expression of the pro-inflammatory cytokine *Il-6* in colonic tissues (Figures 1F,G). IL-6 is known to modulate hepcidin expression and disrupt systemic iron homeostasis, potentially leading to inflammatory anemia (42–44). The mechanisms described above are plausible hypotheses derived from our data and previous research. To comprehensively validate this regulatory pathway, further assessments of iron content in the liver and spleen, as well as hepcidin expression, are required.

Finally, we acknowledge the limitations of the current study as follows: Firstly, the clinical translational value of this research is limited. ADF demonstrates poor long-term adherence and is challenging to implement in asymptomatic individuals at high risk for IBD. Furthermore, this study solely investigated the effects of preventive ADF during the pre-inflammatory stage, without employing active colitis models. Consequently, our findings are not applicable to patients diagnosed with IBD, and the indiscriminate application of ADF is not recommended in clinical settings. Secondly, the effects of ADF are influenced by various confounding factors. The biological impacts of fasting can vary depending on the stage of the disease, nutritional status, and inflammatory conditions. Patients with active IBD often experience malnutrition, and the implementation of fasting interventions may pose potential risks if not applied with caution. For future clinical investigations, it is advisable to consider less stringent dietary regimens, such as time-restricted feeding, in conjunction with professional nutritional monitoring. Thirdly, the use of animal models presents inherent limitations. The acute DSS-induced colitis model primarily replicates short-term acute intestinal injury and does not accurately reflect the pathological characteristics of chronic human IBD, such as recurrent episodes and persistent immune dysfunction. Consequently, our findings are specific to this acute intestinal injury model and should not be directly extrapolated to patients with chronic IBD. Furthermore, in the current study, mice were monitored for a limited duration of 48 h (from day 33 to day 35) following the cessation of DSS treatment. Although certain phenotypes of gastrointestinal injury were observed, the initiation and completion of critical repair mechanisms, including gastrointestinal mucosal repair, intestinal flora reconstruction, and immune function recovery, typically necessitate a more extended observation period. Therefore, the 48-h observation window may not comprehensively capture the entire repair process of the gastrointestinal tract, representing a significant limitation of this study. Fourthly, the mechanistic exploration in this study remains in its preliminary stages. We assessed the effects of ADF primarily based on macroscopic phenotypes. All potential regulatory mechanisms are hypothesized based on existing literature and phenotypic observations, as targeted experiments to verify molecular pathways have not yet been conducted. Additional experimental evidence is necessary to substantiate these mechanistic interpretations.

In conclusion, this study utilizing a preventive DSS-induced colitis mouse model has demonstrated that ADF can moderately exacerbate colitis and its associated anemic complications. This exacerbation is likely due to the impairment of the intestinal mucus barrier, disruption of immune homeostasis, and disturbance of iron metabolism. The findings elucidate the specific effects of preventive ADF on colitis and offer foundational data for further research on dietary interventions targeting intestinal inflammation. Furthermore, our results indicate that patients with IBD should exercise caution when considering ADF as a dietary intervention strategy.

## Data Availability

The original contributions presented in the study are included in the article/Supplementary material, further inquiries can be directed to the corresponding author.
