# Supplementary material for: Alternate-day fasting increases the risk of decreased immune cells and anemia in DSS-induced colitis mice
Source: Front Nutr. 2026 Aug 17;13:1835068. doi: 10.3389/fnut.2026.1835068 (PMC13526943; doi:10.3389/fnut.2026.1835068)
Supplement: Supplementary file 1 [file Data_Sheet_1.pdf]

Supplementary Material

1 Supplementary Figures and Tables

1.1 Supplementary Tables

Supplementary Table 1. The RT-qPCR primers used in this study.

Gene symbol	Forward	Reverse
Il6	TAGTCCTTCCTACCCCAATTTCC	TTGGTCCTTAGCCACTCCTTC
Il17a	GCAACTGTTCCTGAACTCAACT	ATCTTTTGGGGTCCGTCAACT
Tnf-α	CCTGTAGCCCACGTCGTAG	GGGAGTAGACAAGGTACAACCC
Muc2	GCTGACGAGTGGTTGGTGAATG	GATGAGGTGGCAGACAGGAGAC
Claudin1	ATGGTAGCCACTTGCCTTCAG	AGTTCGTCCATTTTTCGGCAG
Occludin	TTGAAAGTCCACCTCCTTACAGA	CCGGATAAAAAGAGTACGCTGG
TJP1	GCCGCTAAGAGCACAGCAA	GCCCTCCTTTTAACACATCAGA
β-actin	GATCATTGCTCCTCCTGAGC	ACTCCTGCTTGCTGATCCAC

Supplementary Table 2. Histopathological scoring of colon HE staining in different experimental groups.

Group	Sample ID	Inflammation/Infiltration Depth Score		Mean Inflammation Depth Score	Crypt Structural Damage Score		Mean Crypt Damage Score	Ulcer Area Score		Mean Ulcer Area Score	Repair Response Score		Mean Repair Response Score	Total Score
Ctrl	1	0	0	0	0	0	0	0	0	0	0	0	0	0
	2	1	0	0.5	0	0	0	0	0	0	0	1	0.5	1
	3	1	1	1	0	0	0	0	0	0	1	1	1	2
DSS	4	1	2	1.5	3	3	3	3	3	3	1	0	0.5	8
	5	2	3	2.5	3	2	2.5	2	2	2	1	1	1	8
	6	1	1	1	3	2	2.5	2	3	2.5	1	1	1	7
ADF+DSS	7	2	3	2.5	4	3	3.5	3	3	3	2	2	2	11
	8	2	3	2.5	3	2	2.5	3	3	3	2	2	2	10
	9	2	3	2.5	4	3	3.5	2	2	2	1	1	1	9

Colon tissues from mice in each group were evaluated by H&E staining and scored based on: (1) Inflammation/infiltration depth; (2) Crypt structural damage; (3) Ulcer/mucosal defect area; (4) Mucosal hyperplasia/repair response.

1.2 Supplementary Figures

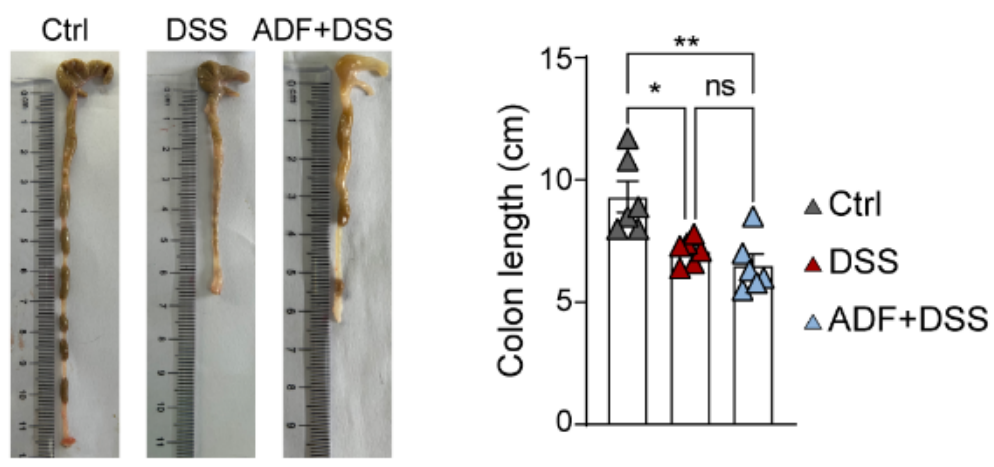

Supplementary Figure 1. Effects of ADF on colon length in mice with DSS-induced colitis. Left, morphology and length (from appendix to anus) of colon tissues. Right, statistics of colon length. Data are representative of at least three independent experiments. Data are presented as mean±SEM and statistical significance was determined by one-way ANOVA with Newman-Keuls multiple comparisons test, **P*<0.05, ***P*<0.01, ns: no significant difference.

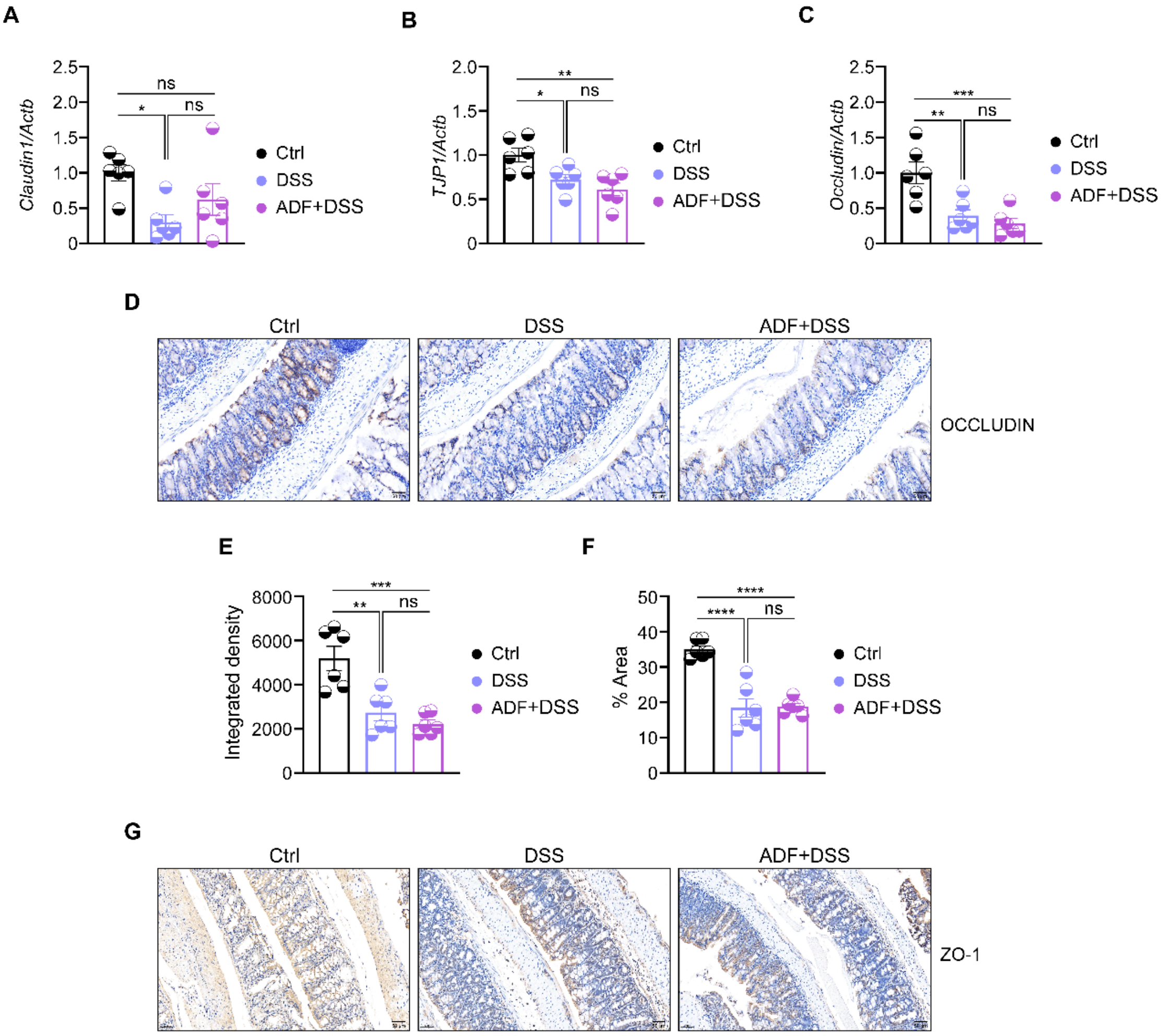

Supplementary Figure 2. Impact of ADF on the expression of intestinal tight junction proteins *Claudin 1*, *TJP1* and *Occludin* of DSS-treated mice. (A-C) RT-qPCR was used to detect the expression of *Claudin 1*, *TJP1* and *Occludin* in colon tissue of mice in each group (n=6/group); **(D)** Immunohistochemistry was used to detect the expression of OCCLUDIN in colon tissue of each group mice. scale bar = 50 μm (n=6/group); **(E-F)** Quantification of immunohistochemical staining for OCCLUDIN (n=6/group); **(G)** Immunohistochemistry was used to detect the expression of ZO-1 in colon tissue of each group mice. scale bar = 50 μm (n=3/group). Data are representative of at least three independent experiments. Data are presented as mean±SEM and

statistical significance was determined by one-way ANOVA with Newman-Keuls multiple comparisons test, ***P*<0.01, ****P*<0.001, *****P*<0.0001, ns: no significant difference.

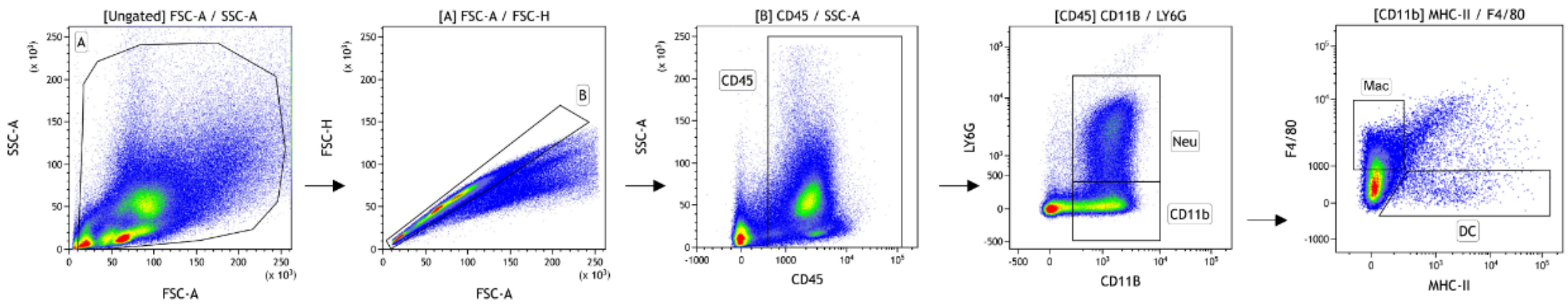

Supplementary Figure 3. Effects of ADF on WBC, neutrophils, macrophage and DC in BM of DSS-treated mice.

The number of WBC, neutrophils and DC in BM of each group mice was analyzed by flow cytometry. CD45 antibody labeled WBC. CD45, CD11b and LY6G antibody labeled neutrophils ($CD45^+CD11b^+LY6G^+$). Macrophage labeled with CD45, CD11b, LY6G, MHC-II and F4/80 antibodies ($CD45^+CD11b^+LY6G^-MHC\text{-}II^-F4/80^+$), DC labeled with CD45, CD11b, LY6G, MHC-II and F4/80 antibodies ($CD45^+CD11b^+ LY6G^-MHC\text{-}II^+F4/80^-$).

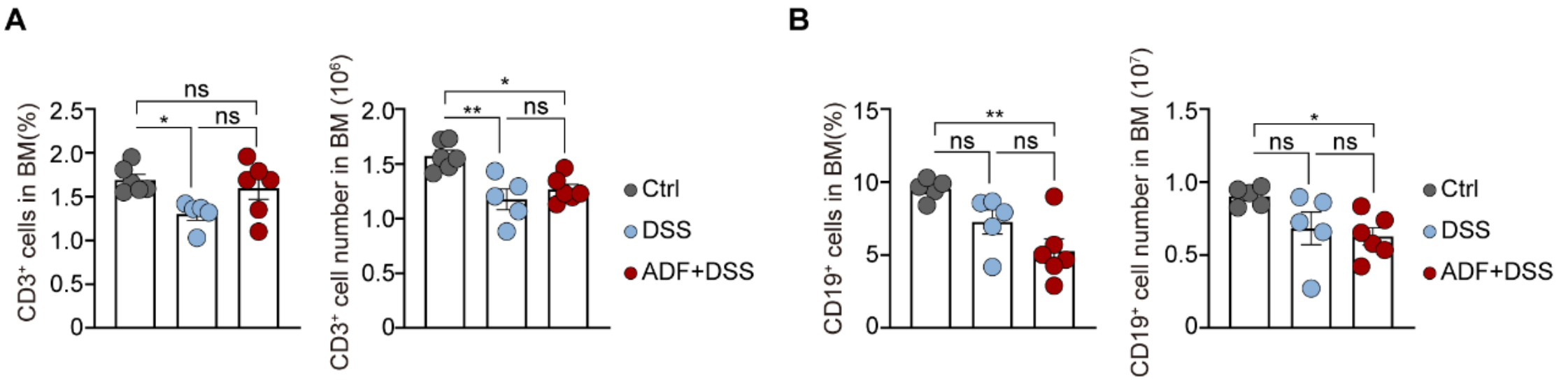

Supplementary Figure 4. T cells and B cells in BM were detected by flow cytometry.

(A) Quantitative and statistical analysis of BM $CD3^+$ T cells (DSS n=5; Ctrl, ADF+DSS n=6); **(B)** Quantitative and statistical analysis of BM $CD19^+$ B cells (Ctrl, DSS n=5; ADF+DSS n=6). Data are representative of at least three independent experiments. Data are presented as mean±SEM and statistical significance was determined by one-way ANOVA with Newman-Keuls multiple comparisons test, **P*<0.05, ***P*<0.01, ns: no significant difference.

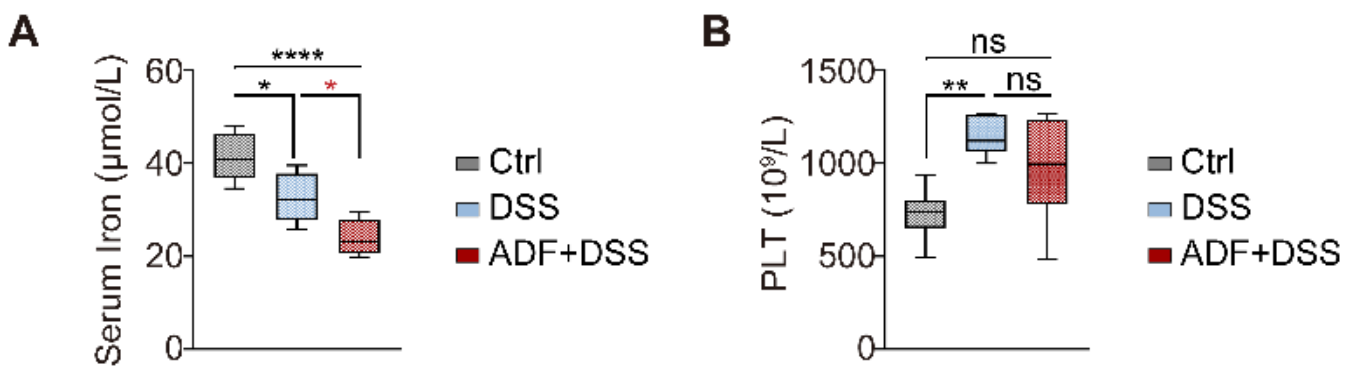

Supplementary Figure 5. Determination of serum iron levels and platelet count

(A) Detection of serum iron levels in each mouse group by ELISA (n=6/group); **(B)** The number of platelets in peripheral blood of mice in each group was detected by routine blood test (n=6/group). Data are representative of at least three independent experiments. Data are presented as mean±SEM and statistical significance was determined by one-way ANOVA with Newman-Keuls multiple comparisons test, $^{*}P<0.05$, $^{**}P<0.01$, $^{****}P<0.0001$, ns: no significant difference.
